# *Loncomelos koprulense* (Asparagaceae), a new species from southern Turkey

**DOI:** 10.3897/phytokeys.175.62037

**Published:** 2021-03-19

**Authors:** Sandro Bogdanović, Salvatore Brullo, Cristina Salmeri

**Affiliations:** 1 University of Zagreb, Faculty of Agriculture, Department of Agricultural Botany, Svetošimunska 25, 10000 Zagreb, Croatia University of Zagreb Zagreb Croatia; 2 Centre of Excellence for Biodiversity and Molecular Plant Breeding, Svetošimunska 25, 10000 Zagreb, Croatia Centre of Excellence for Biodiversity and Molecular Plant Breeding Zagreb Croatia; 3 Department of Biological, Geological and Environmental Sciences, University of Catania, Via A. Longo 19, 95125 Catania, Italy University of Catania Catania Italy; 4 Department of Biological, Chemical and Pharmaceutical Sciences and Technologies (STEBICEF), Palermo University, Via Archirafi 38, 90123 Palermo, Italy Palermo University Palermo Italy

**Keywords:** Distribution, karyology, Mediterranean, *
Ornithogalum s.l.*, Ornithogaleae, taxonomy

## Abstract

A new species, *Loncomelos koprulense* (Asparagaceae), is described and illustrated from southern Turkey. It is a very rare endemic species growing on small semi-rocky escarpments within the Köprülü Kanyon in the province of Antalya. Morphologically for its hairy leaves, *L. koprulense* shows some relationships with *L. malatyanum* and *L. tardum*, species localized in Anatolia too. The chromosome number of the new species is 2*n* = 2*x* = 22. Geographical distribution map for *L. koprulense*, *L. malatyanum* and *L. tardum* is provided.

## Introduction

The genus *Ornithogalum*L., on account of its remarkable morphological and karyological variability, has been the object of various taxonomical treatments, which led to the recognition of several subgenera, sections and series or its splitting into different genera (Rafinesque 1840; Salisbury 1866; Speta 1998a, b, 2001; Pfosser and Speta 1999; Manning et al. 2009; Martínez-Azorín et al. 2009). Recently, phylogenetic investigations based on morphological and molecular approaches carried out by Martínez-Azorín et al. (2011) emphasized that the hierarchical arrangement partly delineated by Speta (1998a) must be pursued, recognizing 19 monophyletic genera within the subfamily Ornithogaloideae Speta, all of which are morphologically well characterized. This approach was followed by Bogdanović et al. (2020) who widely analyzed the taxonomic aspect regarding these groups of Ornithogaleae J.C.Manning and Goldblatt. One of the accepted genera of this tribe, quite widespread in the Mediterranean territories, is *Loncomelos* Raf. showing in particular close relationships with *Ornithogalum*L. s.str. Morphologically, *Loncomelos* is mainly characterized by having inflorescence arranged in an elongated raceme, with pedicels more or less equal at maturity, capsule ovate-lanceolate, trigonous or trilobate with blunt or slightly retuse edges in cross section, seeds polygonal or irregularly compressed, with tuberculate, papillate or rugose testa, while *Ornithogalum* is differentiated by inflorescence corymbose or racemose-corymbose, capsule obovate or oblong, deeply trilobate with six evident ribs in cross section, seeds globose, with sinuous and prominent reticulate testa (Speta 1998a; Martínez-Azorín et al. 2011).

Currently, *Loncomelos* is represented by ca. 32 taxa, formerly mostly attributed to *Ornithogalum*, which are characterized by a very variable chromosome complement differing among the species, from diploid to polyploid and even aneuploid assets with 2n = 14, 16, 18, 20, 22, 24, 26, 28, 32, 36, 42, 44, 46, 52, 54, 88 (Cullen and Ratter 1967; Wittmann 1985; Speta 1998a, 2006, 2010, 2011; Mutlu and Karakuş 2012; Kypriotakis et al. 2018; Bogdanović et al. 2020). In the frame of taxonomic investigation on the genus *Loncomelos*, it is herein examined a very peculiar population collected in the southern Turkey, between Antalya and Manavgat. Based on careful morphological, anatomical and karyological observations, it was concluded that this new geophyte is taxonomically quite isolated, only showing some similarities in its hairy leaves with *L. tardum* Speta and *Ornithogalum malatyanum* Mutlu (here considered as a member of *Loncomelos*), both occurring in Turkey (Speta 2006; Mutlu and Karakuş 2012). Therefore, it is described herein as a new species and named *L. koprulense*.

## Materials and methods

The morphological study on the new species was carried out on living material collected from the locus classicus and cultivated in the Botanical Garden of Catania (Italy). Voucher specimens are deposited in the herbarium CAT (abbreviation follows Thiers 2020). Qualitative and quantitative morphological features were measured and scored on ten fresh plants, using a Zeiss Stemi SV11 Apo stereomicroscope at 6–66× magnification. Morphological comparison with the most related species was carried out using literature data (Speta 2006; Mutlu and Karakuş 2012). The diagnostic traits of the new species and its two allied ones are shown in Table 1.

**Table 1.** Main morphological differences among *Loncomelos koprulense*, *L. tardum* and *L. malatyanum*.

Characters	*L. koprulense*	*L. tardum*	*L. malatyanum*
Plant tall (cm)	up to 95	up to 80	up to 73
Bulb shape	subglobose	ovoid	ovoid-globose
Bulb size (cm)	2.5–3 × 3–3.6	2.5–4 × 2.5	2.5–2.7 × 1.5–2.5
Bulb tunic colour	whitish	grey-brown	whitish
Scape height (cm)	55–60	40–65	34–53
Leaf number	4	3–4	5–7
Leaf length (cm)	18–30	up to 35	(25) 28–40
Leaf width (mm)	3.5–8	4–5	3–10 (11)
Inflorescence length (cm)	32–40	23–28	12–20
Number of flowers	50–55	40	18–30 (55)
Flower pedicel length (mm)	12–25	14–25	5–15
Bract shape	ovate-lanceolate	subulate	subulate
Bract length (mm)	6–13(19)	10–16	7–18
Bract margin	smooth	smooth	0–1 (2) toothed
Bract / pedicel ratio	shorter to subequal	about a half	equal or longer
Tepal size (mm)	10–11 × 2.4–2.6	9.5–11.3 × 1.7–2.6	9–12 × 2.2–4
Tepal shape	linear-oblong	linear	lanceolate to elliptical
Tepal colour	green, with white margin	greenish, with white margin	whitish, green in the centre
Tepal margin	undulate	slightly rolled, flat	flat
Staminal filament shape	oblong, narrowed and apiculate at the apex	oblong, apiculate at the apex	lanceolate, acuminate at the apex
Staminal filament size (mm)	5–5.5 × 1.6–2	6 × 1.7–1.9	5.5–6 × 1.6
Anther length (mm)	2.5–2.7	2.8	2.2–3.2
Anther colour	pale–green	greenish	yellowish-light green
Ovary shape	ovoid	ovoid	cylindrical
Ovary size (mm)	3 × 2.3	3–3.5 × 2.2–2.5	2–3.3 × 2.2–2.8
Style length (mm)	2.2–2.3	3–3.8	4–5.3
Capsule shape	ovoid	ellipsoid	ovoid to globose
Capsule size (mm)	6.5–7 × 5	8–9 × 6	(5) 7–11 × (4) 5–7
Chromosome number (2n)	22	20	24

Leaf anatomy was studied on cross-sections from cultivated plants, using fresh blades of minimum sized and maximum sized leaves in their optimal vegetative phase.

Karyological analyses were performed on root tip cells of cultivated bulbs, pre-treated with a 0.3% (w/v) colchicine water solution for 3 h at room temperature, fixed in Farmer’s fixative (3:1 v/v, absolute ethanol: glacial acetic acid) for 12 h and stored in 70% ethanol water solution. Then, root tips were hydrolysed in 1 N HCl for 7 min at 60 °C and stained according to the Feulgen technique. Microphotographs of at least 10 good metaphase plates from different individuals were taken using a Zeiss PrimoStar microscope equipped with a Canon PowerShot G9 digital camera. Metaphase chromosomes were measured by the Zeiss Axiovision 4.8 image analysis software, while karyotyping was performed by CROMOLAB 1.1 software (Brullo 2002). The chromosome types were named according to the centromere position based on Levan et al. (1964) and Tzanoudakis (1983). All measured karyomorphometric parameters are provided in Table 2.

**Table 2.** Karyomorphometric parameters and karyotype symmetry indices of *Loncomelos koprulense*. Values come from 10 good metaphase plates from individuals of the type localities.

Chromosome	TAL (µm)			TRL%			AR	CI	Type
pairs	Mean ± SD	Max	Min	Mean ± SD	Max	Min			
I	10.8 ± 1.3	12.3	9.2	7.0 ± 0.7	7.9	6.1	1.06	48.6	m
II	10.0 ± 1.2	11.8	8.9	6.5 ± 0.3	6.6	6.4	1.13	46.9	m
III	8.8 ± 1.0	10.1	7.4	5.7 ± 0.2	6.8	6.0	1.22	45.1	m
IV	8.8 ± 1.1	10.0	7.4	5.7 ± 0.4	6.4	5.2	1.49	40.2	msm
V	6.9 ± 1.3	9.0	5.1	4.5 ± 0.4	5.1	3.8	2.49	28.6	sm
VI	6.5 ± 1.3	7.8	4.9	4.2 ± 0.6	5.1	3.6	1.39	41.8	msm
VII	5.9 ± 0.6	6.7	5.3	3.9 ± 0.3	4.4	3.5	2.69	27.1	sm
VIII	5.4 ± 0.8	6.7	4.5	3.5 ± 0.1	3.8	3.3	2.17	31.5	sm
IX	4.9 ± 0.6	5.7	4.2	3.2 ± 0.1	3.4	3.1	2.38	29.6	sm
X	4.5 ± 0.3	4.9	4.0	2.9 ± 0.2	3.3	2.6	1.80	35.7	sm
XI	4.5 ± 0.9	5.7	3.4	2.9 ± 0.5	3.4	2.2	1.38	42.0	msm
**TCL**: 153.9 ± 17.1 µm; **MCL**: 7.0 ± 2.2 µm; **d-value**: 32.5; **DRL**%: 4.5; **S**%: 38.3; **MAR**: 1.54; **MCI**: 38.7; **Cv_CL_**: 32.2; **Cv_CI_**: 21.2; **M_CA_**: 24.2; **Stebbins’ category**: 2B									

**Abbreviations**: TAL = total absolute length; TRL = total relative length; AR = arm ratio index; CI = centromeric index; Type = chromosome nomenclature; TCL = total chromosome length; MCL = mean chromosome length; d-value = difference between long arms and short arms; DRL% = difference of relative length; S% = relative length of shortest chromosome; MAR = mean arm ratio index; MCI = mean centromeric index; Cv_CL_ = coefficient of variation of chromosome length; Cv_CI_ = coefficient of variation of centromeric index; MCA = mean centromeric asymmetry.

## Taxonomy

### Loncomelos koprulense

Taxon classificationPlantaeAsparagalesHyacinthaceae

Bogdanović, Brullo & Salmeri sp. nov.

D9278E22-3261-5F1C-B49C-B9D16A96DAB5

urn:lsid:ipni.org:names:77215897-1

Figs 1 , 2 , 5

#### Type.

Turkey. Antalya: District of Manavgat, Köprülü Kanyon National Park, Bozyaka road, cultivated specimen, 15 June 2010, *Brullo s.n.* (Holotype: CAT!).

#### Diagnosis.

Loncomelo malatyano affine, sed distinctum statura majore, bulbo subgloboso, latiore, 4–foliato, inflorescentia longiore, 50–55 floribus, bracteis ovato-lanceolatis, non dentatis, tepalis lineari-oblongis, viridibus, albis et undulatis margine, filamentis staminorum oblongis, latioribus, ovario ovoideo, stylo breviore, capsula minore.

**Figure 1.**
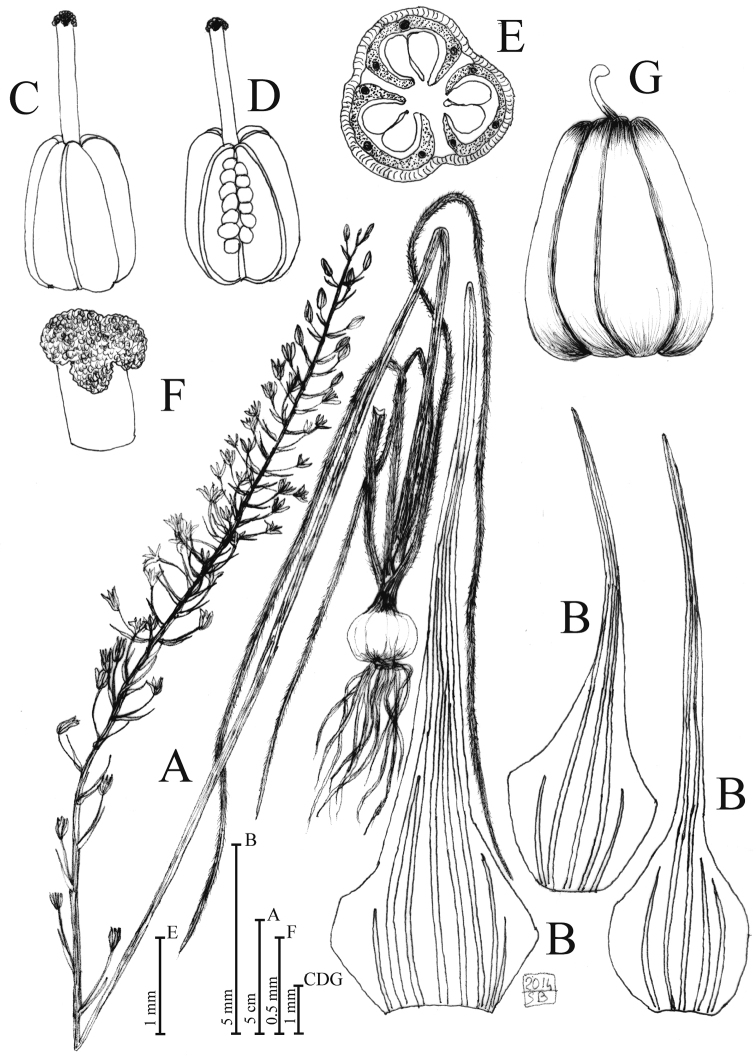
*Loncomelos koprulense* Bogdanović, Brullo & Salmeri, sp. nov. **A** habit **B** bracts **C** pistil **D** open pistil **E** ovary cross section **F** stigma **G** capsule. Drawing by S. Brullo from cultivated material coming from the type locality.

#### Description.

Plant up to 95 cm tall. Bulbs subglobose, 2.5–3 × 3–3.6 cm, outer tunics whitish, papery, without bulblets. Scape 55–60 cm long, glabrous, green-glaucous. Leaves 4, often withered at the anthesis, shorter than scape, linear, canaliculate, 18–30 × 0.35–0.8 cm, without white median line, abaxial face densely hairy, margins hairy, hairs 0.5–1.2 mm long, adaxial one glabrous. Raceme cylindrical, 32–40 cm long, 50–55 flowered. Pedicels 12–25 mm long, curved-divaricated, glabrous. Bracts membranous, ovate-lanceolate, 6–13(19) mm long, broadened at the base, long acuminate toward the apex, 5–8 nerved, shorter than pedicel or subequal, smooth at the margin, glabrous. Perigon stellate, 20 mm in diameter, tepals linear-oblong, 10–11 × 2.4–2.6 mm, glabrous, papillate-glandulose at the apex, markedly undulate at the margin, green with white margin. Staminal filaments white, oblong, abruptly narrowed and apiculate at the apex, 5–5.5 × 1.6–2 mm, thickened at the margin with central greenish midrib, anthers pale-green, 2.5–2.7 mm, dorsifixed. Ovary ovoid, green, 3 × 2.3 mm, with blunt lobes; each lobe with a distinct central nerve and two smaller, lateral ones. Style 2.2–2.3 mm long, stigma papillose. Capsule ovoid, 6.5–7 × 5 mm, erect. Seeds not seen. Chromosome number 2*n* = 2*x* = 22.

**Figure 2.**
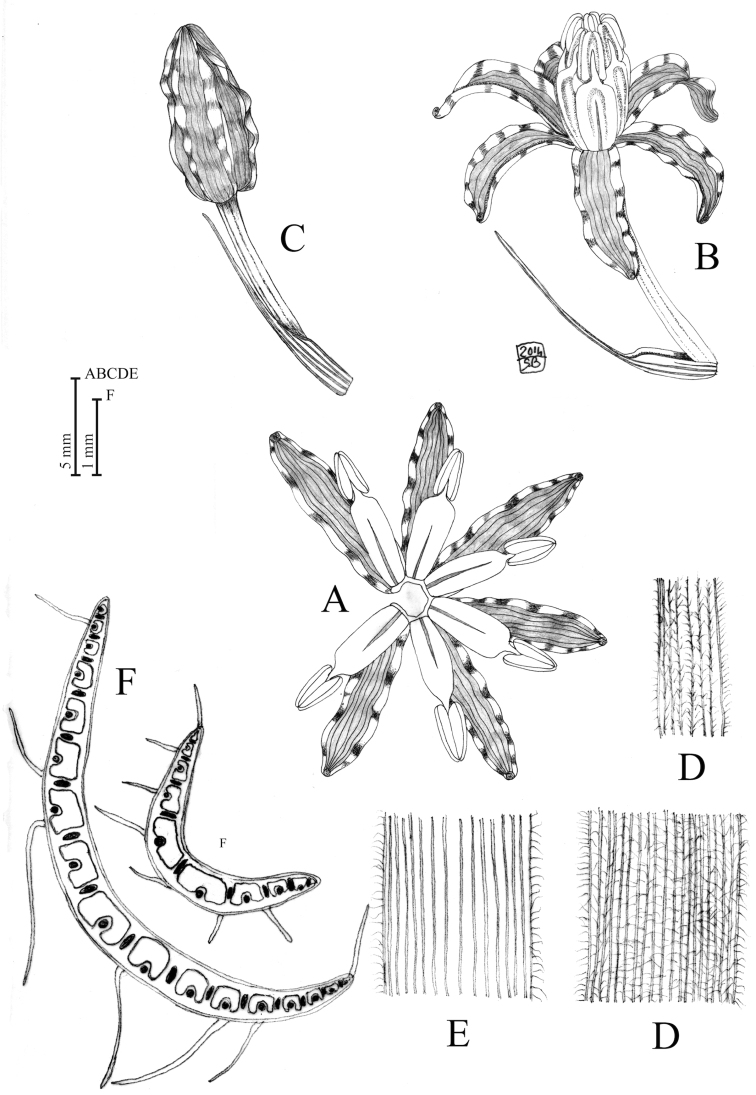
*Loncomelos koprulense* Bogdanović, Brullo & Salmeri, sp. nov. **A** tepals and stamens **B** flower with bract **C** bud with bract **D** leaf abaxial face **E** Leaf adaxial face **F** leaf cross sections. Drawing by S. Brullo from cultivated material coming from the type locality. Darker strips in tepal edges mark the undulations.

#### Phenology.

Flowering in June and fruiting in June-July.

#### Etymology.

The species epithet is derived from the name of the Köprülü Kanyon, locality where this geophyte was collected.

#### Karyology.

**Figure 3.**
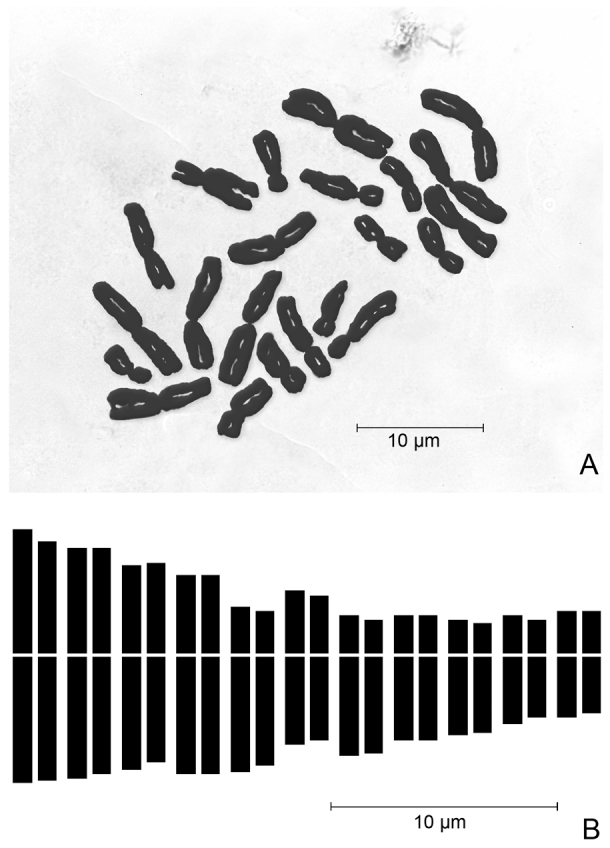
Chromosome complement (2*n* = 2*x* = 22) of *Loncomelos koprulense***A** mitotic metaphase plate from the type locality **B** idiogram.

All investigated samples of *Loncomelos koprulense* from the type locality revealed a somatic chromosome complement with 2*n* = 22 (Fig. 3A). The karyotype is rather asymmetrical, comprising 11 chromosome pairs (Fig. 3B), arranged in two size groups where the submedian type prevails, as highlighted by the values of different symmetric indices (Table 2). In particular, there are 3 metacentric pairs, 3 meta-submetacentric pairs (showing arm ratio exceeding 1.30), and 5 submetacentric pairs (3 big-sized and 2 small-sized). Thus, the chromosome formula can be expressed as 2*n* = 2x = 22 = 6 m + 6 msm + 10 sm. No evident satellites were detected. Absolute chromosome length varied from 11.1 ± 1.3 μm of the longest chromosome and 4.26 ± 0.3 μm of the shortest one, with a mean chromosome length of 6.99 ± 2.2 μm. Relative chromosome length varied from 7.24% ± 0.8 to 2.78% ± 0.2. Arm index varied on average from 1.03 to 2.76, while the centromeric index ranged from 49.3 to 26.6. All karyomorphometric parameters are given in Table 2.

#### Leaf anatomy.

The known *Loncomelos* species are usually differentiated by their canaliculate leaves, uniformly colored with dorsi-ventral arrangement, presenting differences in size in the same individual. In particular, the leaf outline is smooth in adaxial faces and more or less ribbed in the abaxial one, with epidermal cells covered by a thickened cuticle; the pallisade tissues is one-layered and distributed along the whole perimeter, while the inner part is occupied by the spongy tissue (Wittmann 1985; Tornadore 1985, 1986; Tornadore and Orza 1987; Lynch et al. 2006; Peruzzi et al. 2007; Öztürk et al. 2014; Bogdanović et al. 2020). The vascular bundles are arranged in two rows all along the mesophyll; large vascular bundles occur in the central part, which are alternated with other smaller one towards the abaxial side. The large bundles are interspersed with mucilage cells that are replaced by rhexigenetic lacunae in the mature leaves. Most species have completely glabrous leaves, except for *L. tardum*, *L. malatyanum* (see below for nomenclatural validation) and *L. koprulense*, showing a dense hairiness on the abaxial face. As a whole, the leaves of *L. koprulense* maintain the main features of the genus, revealing a marked variability in size; the largest leaves are characterized by 17–18 large vascular bundles, interposed among lacunae; these bundles decrease in number in the progressively narrower leaves up to a minimum of ca. 9, while the number of small vascular bundles coincides with that of the mesophyll lacunae (Fig. 2F). As far as hairs are concerned, they are irregularly distributed along the margin and on the abaxial face.

#### Ecology and distribution.

**Figure 4.**
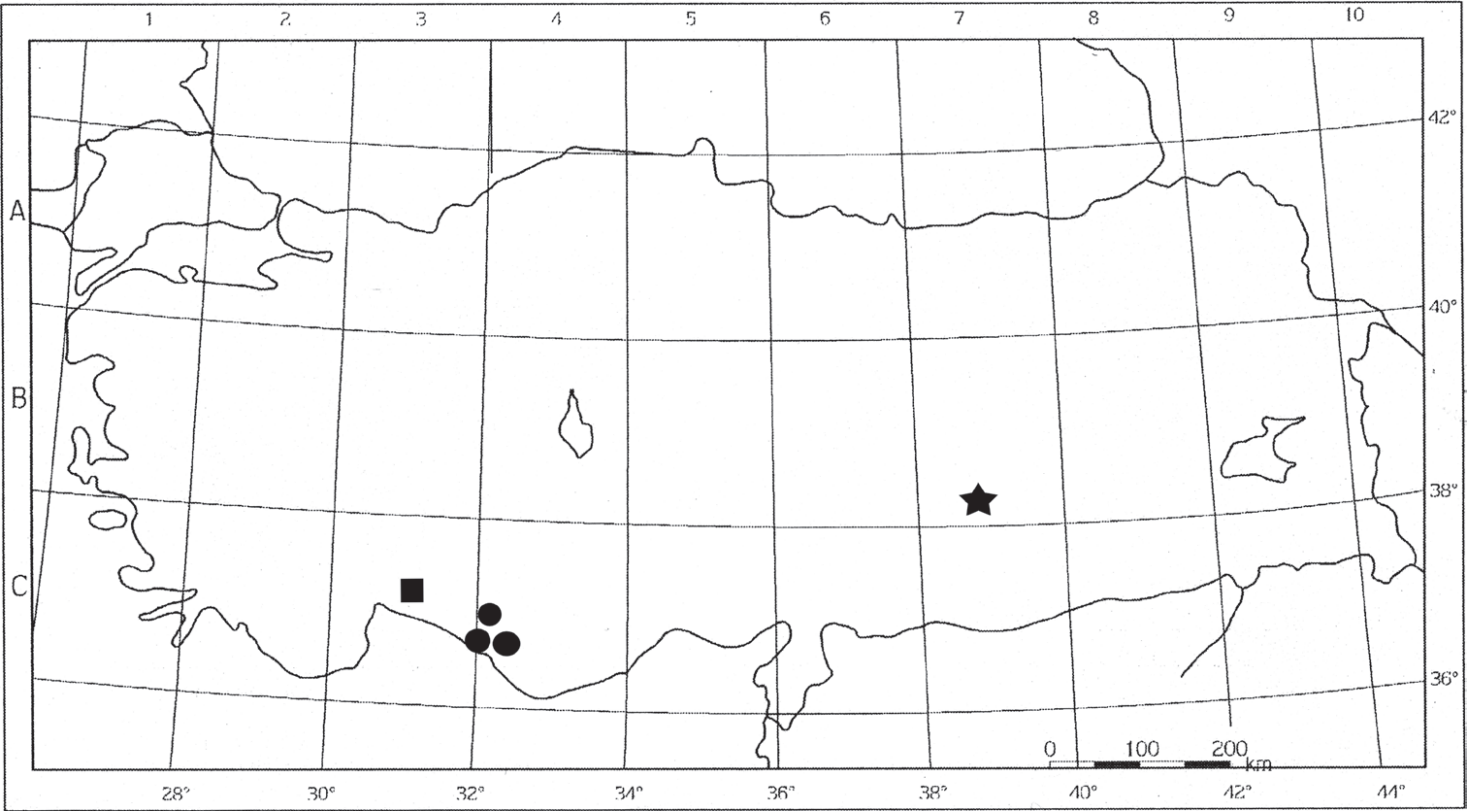
Distribution map of *Loncomelos koprulense* (square), *L. tardum* (circle) and *L. malatyanum* (star).

*Loncomelos koprulense* seems to be a very rare species currently know only for a single locality of southern Turkey. One small and well circumscribed population was surveyed along the Bozyaka road within the Köprülü Kanyon at about 150 m of elevation (Fig. 4), where it grows on small semi-rocky escarpments covered by a scarce herbaceous vegetation. The woody vegetation near this habitat is represented by a thermophilous maquis characterized by *Quercus calliprinos* Webb, Olea europaea L. subsp. sylvestris (Mill.) Rouy ex Hegi, *Pistacia terebinthus*L., *Juniperus oxycedrus*L., *Myrtus communis*L., *Arbutus andrachne*L., *Cupressus sempervirens*L. etc. (Tavşanoğlu and Coşkun 2009). This area, falling within an important National Park and known as Köprülü Kanyon Milli Parkı between Antalya and Manavgat, is floristically highly rich in endemic species as emphasized by Özçelik (2018).

#### Additional examined material.

Turkey. Antalya: District of Manavgat, Köprülü Kanyon National Park, Bozyaka road, cultivated specimen, 24 June 2013, *Brullo s.n.* (paratype: CAT!).

#### Discussion.

From the literature data (Zahariadi 1977, 1980; Wittmann 1985; Martínez-Azorín 2008; Martínez-Azorín et al. 2009), the circumscription of the genus *Ornithogalum* within the tribe Ornithogaleae has always been problematic, emphasizing that the traditional morphological approach is not sufficient to discriminate the taxa at generic level. Recent phylogenetic studies carried out by Pfosser and Speta (1999) and Martínez-Azorín et al. (2011), based on cpDNA and nrDNA gene sequences, have provided a relevant support for a taxonomic arrangement of this tribe, validating the treatment previously proposed by Speta (1998a, b). As concern the genus *Loncomelos*, it is morphologically well differentiated from *Ornithogalum* s.str. by numerous and significant characters regarding the inflorescence, pedicel, capsule and seed. From the phytogeographical point of view, this genus is mainly distributed in the Mediterranean area with the higher concentration of species in the Balkan Peninsula and Anatolia. The last territory currently hosts 14 species (included the new one), that therefore can be considered the main centre of differentiation of the genus.

**Figure 5.**
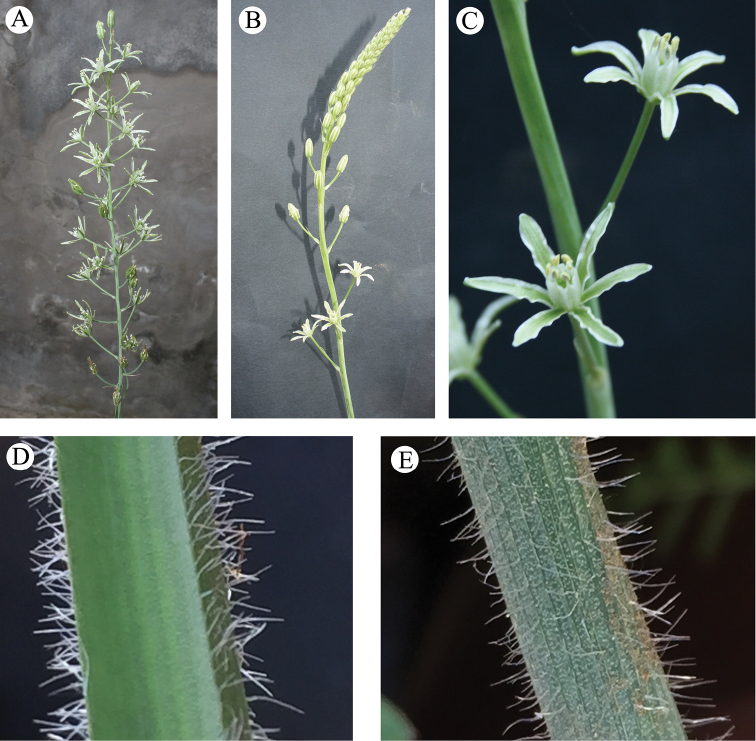
*Loncomelos koprulense***A, B** inflorescence **C** flower **D** leaf abaxial face **E** leaf adaxial face from the type locality.

A very peculiar and significant morphological character occurring in *Loncomelos koprulense* is the densely hairy leaves (Fig. 5D–E). In fact, most species of the genus *Loncomelos* are characterized by glabrous leaves, while only *L. tardum* and *L. malatyanum* have hairs on the leaves. According to Speta (2006) and Mutlu and Karakuş (2012), both species occur in Anatolia too, where they are very rare and quite localized (Fig. 4). They differ from *L. koprulense* in some relevant morphological features (Table 1), such as the shape and size of the bulbs, number of leaves, inflorescence size, number of raceme flowers, bracts, tepal shape and colour, tepal margin, stamen filament, size and shape of ovary and capsule. Differences were also observed in karyological aspect, since *L. tardum* is characterized by a chromosome complement of 2*n* = 2*x* = 20, reported by Speta (2006), while according to Mutlu and Karakuş (2012) the chromosome number of *L. malatyanum* is 2*n* = 2*x* = 24. We found out that *L. koprulense* is also a diploid, but its chromosome number is 2*n* = 2*x* = 22, which is quite rare in the genus *Loncomelos*, so far only previously counted in *L.fischerianum* (Krasch.) Speta by Agapova (1977).

#### Nomenclatural note.

*Loncomelos malatyanum* (Mutlu) Bogdanović, Brullo & Salmeri comb. nov. ≡ *Ornithogalum malatyanum* Mutlu in Mutlu and Karakuş Turkish Journal of Botany 36: 126 (2012), basionym.

## Supplementary Material

XML Treatment for Loncomelos koprulense

## References

[B1] Agapova ND (1977) Cytosystematic investigation of the European representatives of the genus *Ornithogalum* (Fam. Liliaceae) of the U.S.S.R. flora I. Bot. Zurn. 62: 970–983.

[B2] Bogdanović S Brullo S Ljubičić I Rat M Salmeri C (2020) Cytotaxonomical remarks on *Loncomelos visianicum* (Hyacinthaceae), a poorly known species endemic to Croatia. Phytotaxa 430: 095–108. 10.11646/phytotaxa.430.2.2

[B3] Brullo F (2002) Cromolab. Dipartimento di Botanica Università degli Studi di Catania.

[B4] Cullen I Ratter JA (1967) Taxonomic and cytological notes on Turkish *Ornithogalum*. Notes from the Royal Botanic Garden Edinburgh 27(3): 293–339.

[B5] Kypriotakis Z Antaloudaki E Tzanoudakis D (2018) *Ornithogalum insulare* (Hyacinthaceae): A new species from the Cretan area (S. Aegean, Greece). Botanica Serbica 42: 117–122.

[B6] Levan A Freda K Sandberg AA (1964) Nomenclature for centromeric position on chromosomes. Hereditas 52(2): 201–220. 10.1111/j.1601-5223.1964.tb01953.x

[B7] Lynch A Rudall PJ Cutler DF (2006) Leaf anatomy and systematics of Hyacinthaceae. Kew Bulletin 61: 145–159.

[B8] Manning JC Forest F Devey DS Fay MF Goldblatt P (2009) A molecular phylogeny and a revised classification of Ornithogaloideae (Hyacinthaceae) based on an analysis of four plastid DNA regions. Taxon 58(1): 77–107. 10.1002/tax.581011

[B9] Martínez-Azorín M (2008) Sistemática del género *Ornithogalum*L. (Hyacinthaceae) en el Mediterráneo occidental: implicaciones taxonómicas, filogenéticas y biogeográficas. Doctoral Thesis, Universidad de Alicante, Spain.

[B10] Martínez-Azorín M Crespo MB Juan A (2009) Taxonomic revision of Ornithogalum subg. Beryllis (Hyacinthaceae) in the Iberian peninsula and the Balearic islands. Belgian Journal of Botany 142: 139–161.

[B11] Martínez-Azorín M Crespo MB Juan A Fay MF (2011) Molecular phylogenetics of subfamily Ornithogaloideae (Hyacinthaceae) based on nuclear and plastid DNA regions, including a new taxonomic arrangement. Annali di Botanica 107(1): 1–37. 10.1093/aob/mcq207 PMC3002468 21163815

[B12] Mutlu B Karakuş Ş (2012) A new species of *Ornithogalum* (Hyacinthaceae) from East Anatolia, Turkey. Turkish Journal of Botany 36: 125–133.

[B13] Özçelik H (2018) Flora inventory of Köprülü Kanyon National Park (Antalya-Isparta). Turk J Forst. 19: 40–50. 10.18182/tjf.338944

[B14] Öztürk D Koyuncu O Koray Yaylacı Ö Özgişi K Sezer O Tokur S (2014) Comparative anatomical studies on twelve *Ornithogalum* (Asparagaceae) species (eleven nonendemic, one endemic) belonging to subgen. Ornithogalum and subgen. Beryllis, growing naturally in Eskişehir (Central Anatolia-Turkey). International Journal of Scientific Research and Reviews 3: 40–49.

[B15] Peruzzi L Caparelli KF Cesca G (2007) Contribution to the systematic knowledge of the genus *Ornithogalum*L. (Hyacinthaceae): Morpho-anatomical variability of the leaves among different taxa. Bocconea 21: 257–265.

[B16] Pfosser M Speta F (1999) Phylogenetics of Hyacinthaceae Based on Plastid DNA Sequences. Ann Miss Bot Gard. 86(4): 852–875. 10.2307/2666172

[B17] Rafinesque CS (1840) Autikon Botanikon, Centuria V. Philadelphia, 55–71.

[B18] Salisbury RA (1866) The genera of plants: a fragment containing part of Liriogamae. John van Voorst, London, 143 pp.

[B19] Speta F (1998a) Hyacinthaceae. In: Kubitzki K (Ed.) The families and genera of vascular plants 3.Springer, Berlin, 261–285. 10.1007/978-3-662-03533-7_35

[B20] Speta F (1998b) Systematische analyse der Gattung *Scilla*L. s.l. (Hyacinthaceae). Phyton 38: 1–141.

[B21] Speta F (2001) Die Echte und die Falsche Meerzwiebel: *Charybdis* Speta und *Stellarioides* Medicus (Hyacinthaceae), mit Neubeschreibungen und Neukombinationen im Anhang. Stapfia 75: 139–176.

[B22] Speta F (2006) Die Gattung *Loncomelos* Raf. (Hyacinthaceae-Ornithogaloideae), vorgestellt anhand dreier neuer Arten. Phyton 46: 1–25.

[B23] Speta F (2010) Beitrag zur Kenntnis der *Loncomelos narbonensis*-Verwandtschaft (Hyacinthaceae – Ornithogaloideae). Verh Zool-Bot Ges Österreich 147: 125–157.

[B24] Speta F (2011) A remarkable new *Loncomelos* species from NE-Turkey: *L. erichpaschei* Speta spec. nova (Hyacinthaceae-Ornithogaloideae). Phyton 51: 153–160.

[B25] Tavşanoğlu Ç Coşkun U (2009) Effect of goat browsing on growth form of maquis species in Köprülü Kanyon National Park (Antalya, Turkey). Ekoloji 18: 74–80.

[B26] Thiers B (2020) Index Herbariorum: A global directory of public herbaria and associated staff. New York Botanical Garden’s Virtual Herbarium. http://sweetgum.nybg.org/ih/ [accessed 10 February 2020]

[B27] Tornadore N (1985) Il gen. *Ornithogalum*L. (Liliaceae). V. Osservazioni sulla citosistematica di *O. pyrenaicum*L. Atti della Società Toscana di Scienze Naturali – Memorie Serie B 92: 247–257.

[B28] Tornadore N (1986) Il genere *Ornithogalum*L. (Liliaceae). VI. *O. narbonense*L. Atti della Società Toscana di Scienze Naturali – Memorie Serie B 93: 111–120.

[B29] Tornadore N Orza P (1987) Il gen. *Ornithogalum*L. (Liliaceae) in Italia. VIII. Il subgen. Beryllis (Salisb.) Baker con particolare riguardo ad *O. brevistylum* Wolfner. Atti della Società Toscana di Scienze Naturali – Memorie Serie B 94: 341–356.

[B30] Tzanoudakis D (1983) Karyotypes of ten taxa of Allium section Scorodon from Greece. Caryologia 36(3): 259–284. 10.1080/00087114.1983.10797667

[B31] Wittmann H (1985) Beitrag zur Systematik der *Ornithogalum* – Arten mit verlängert-traubiger Infloreszenz. Stapfia 13: 1–117.

[B32] Zahariadi C (1977) Notes on the intrageneric classification of the genus *Ornithogalum*L. (Liliaceae). Botanicheskii Zhurnal 62: 1624–1639. [in Russian]

[B33] Zahariadi C (1980) *Ornithogalum*L. In: Tutin TG Heywood VH Burges NA Moore DM Valentine DH Walters SM (Eds) Flora Europaea 5.Cambridge University Press, Cambridge, 35–40.

